# Development and validation of a nomogram to predict cancer-specific survival of elderly patients with unresected gastric cancer who received chemotherapy

**DOI:** 10.1038/s41598-024-59516-3

**Published:** 2024-04-19

**Authors:** Qi Wang, Kexin Shen, Bingyuan Fei, Mengqiang Wei, Xinbin Ge, Zhongshi Xie

**Affiliations:** Department of Gastrointestinal Colorectal and Anal Surgery, China-Japan Union Hospital of Jilin University, Changchun, China

**Keywords:** Gastric cancer, Chemotherapy, Nomogram, SEER database, Elderly patients, Cancer, Gastrointestinal cancer, Gastric cancer

## Abstract

This investigation aimed to explore the prognostic factors in elderly patients with unresected gastric cancer (GC) who have received chemotherapy and to develop a nomogram for predicting their cancer-specific survival (CSS). Elderly gastric cancer patients who have received chemotherapy but no surgery in the Surveillance, Epidemiology, and End Results Database between 2004 and 2015 were included in this study. Cox analyses were conducted to identify prognostic factors, leading to the formulation of a nomogram. The nomogram was validated using receiver operating characteristic (ROC) and calibration curves. The findings elucidated six prognostic factors encompassing grade, histology, M stage, radiotherapy, tumor size, and T stage, culminating in the development of a nomogram. The ROC curve indicated that the area under curve of the nomogram used to predict CSS for 3, 4, and 5 years in the training queue as 0.689, 0.708, and 0.731, and in the validation queue, as 0.666, 0.693, and 0.708. The calibration curve indicated a high degree of consistency between actual and predicted CSS for 3, 4, and 5 years. This nomogram created to predict the CSS of elderly patients with unresected GC who have received chemotherapy could significantly enhance treatment accuracy.

## Introduction

Gastric cancer (GC) is the fifth most prevalent malignant neoplasm worldwide and the third leading cause of cancer-related death^1^. GC is a condition that predominantly affects the elderly population, with the majority of cases manifesting in those aged ≥ 65 years, reaching its highest incidence at approximately 70 years of age^2,3^. Despite the ongoing advancements in diagnostic and treatment techniques for GC in recent years, which have led to improved prognoses for some patients, the survival rate of GC patients, especially within the elderly population (aged ≥ 65 years), persists at an unsatisfactory level^4^. Currently, the standard treatment strategy for GC involves a multidisciplinary surgical approach^5,6^. As a result of the inconspicuous nature of gastric cancer symptoms, a majority of elderly individuals often find themselves diagnosed at an advanced stage of the disease upon initial examination. Additionally, the elderly population exhibits comparatively poorer health status and a higher prevalence of comorbidities in contrast to their younger counterparts, further complicating matters and often precluding these patients from undergoing surgical interventions. For elderly patients with gastric cancer (EGC)who have lost the chance to undergo surgery, chemotherapy, as an appropriate option, increases the probability of survival, lessens their symptoms, and enhances their quality of life^7^. EGC patients who have not undergone surgery but have received chemotherapy should be seriously considered by clinicians.

For patients with non-operated EGC who have undergone chemotherapy, personalized treatment approaches are deemed more effective than strictly adhering to publicly appropriate treatment principles. Accurately predicting the prognosis is crucial for designing effective treatment plans for this particular population. Currently, GC treatment and prognosis are based on the tumor-node-metastasis (TNM) staging system of the American Joint Committee on Cancer (AJCC)^8^. However, patients having identical TNM staging exhibit substantial variations in prognosis^9,10^. Given the complex characteristics of non-operated elderly patients with EGC who have undergone chemotherapy, it is imperative to devise a novel model that can effectively predict their prognosis. Nomograms have been extensively used as clinical prediction models; they combine many variables to determine the probability of a specific clinical occurrence^11^. The utilization of nomograms in clinical practice can aid surgeons in providing prognostic information regarding survival odds for such populations, thereby enabling the development of personalized treatment strategies for this specific cohort.

In this study, independent CSS-related factors in patients with non-operated EGC who have undergone chemotherapy were analyzed using a group of multicenter cases from the SEER database. Besides, a nomogram was created to estimate their cancer-specific survival (CSS) at 3, 4, and 5 years. This nomogram has the potential to considerably improve the quality of communication between patients with EGC and clinicians, and may help doctors in developing tailored treatment plans based on the particular condition of these patients.

## Methods

### Study population

**Figure 1:**
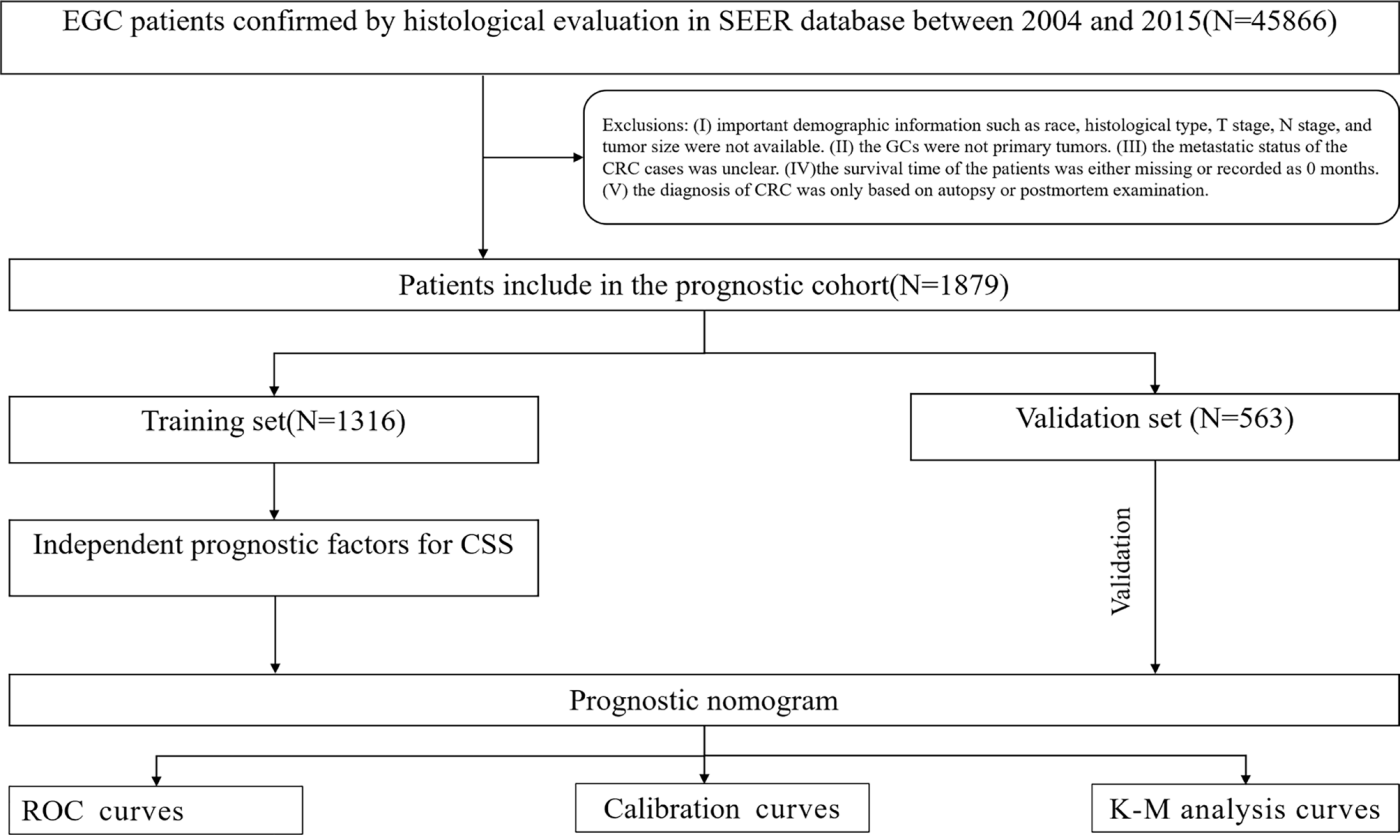
The patient’s selection process and other crucial steps taken throughout this study.

Information on non-operated patients with EGC who had undergone chemotherapy and whose follow-up records were fully complete was obtained from the SEER database using SEER Stat 8.3.6 (www.seer.cancer.gov/seerstat). Patient selection was conducted using the criteria outlined in the 3rd edition of the International Classification of Diseases for Oncology (ICD‐O‐3) based on the primary location of the tumor (C16.0–C18.6, C16.8, C16.9). The exclusion criteria were as follows: (1) crucial details such as tumor size, grade, histology type, or demographic information were not available; (2) GC is not the primary tumor; (3) patients have undergo surgery or unable to receive chemotherapy; (4) the survival time of the patients was either missing or recorded at 0 month; (5) the diagnosis of GC was based only on autopsy or post-mortem examination. Because the data in the SEER database were anonymous, no ethical committee approval or informed permission was required. The process of identifying the patient and other major steps in the study are depicted in Fig. 1.

### Variables selection

We have selected 12 variables to assess the prognosis of our participants, including age at diagnosis, race, sex, marital status, tumor size, histology, grade, T stage, N stage, M stage, radiotherapy, and primary site. Given the absence of universally accepted guidelines for categorizing the age of patients with GC, we have defined the term “EGC” to encompass individuals aged 65 years or older, relying on existing research findings^12,13^. Individuals are categorized into specific racial groups, namely black, white, and others, based on their diverse ethnic backgrounds. The patients were classified into four distinct categories based on the anatomical location of the tumor: (I) gastric cardia, (II) fundus, (III) gastric body, (IV) gastric antrum, (V) pylorus, and (VI) other parts. Age and tumor size were also separated based on the appropriate cut-off value generated by the X-tile software version 3.6.1 (Yale University School of Medicine, US).

### Statistical analyses

Categorical variables were commonly stated using numbers and percentages (N, %); quantitative variables were marked by mean ± standard deviation (SD). All statistical analyses in the current investigation were conducted using SPSS 27.0 (version 27.0) and R software 4.2.2(https://www.r-project.org/). Statistical significance was set at *p* value is ≤ 0.05 (both sides). To ensure that our model was as accurate as possible, we used R software to randomly split the study data into two subsets: a training cohort and a validation cohort (sample ratio, 7:3). Univariate COX regression analysis was conducted on the aforementioned factors in this specific patient population. Variables that exhibited a significant level (*p* < 0.05) in the univariate analysis were subsequently included in the multivariate COX regression analysis, which ultimately determined the independent prognostic variables for non-operated patients with EGC who had undergone chemotherapy (*p* < 0.05). Additionally, to show the extent to which a given prognostic factor affected the CSS, we used the hazard ratio (HR) and the corresponding 95% confidence intervals (CI). Ultimately, relying on the already established independent prognostic factors, the “RMS” program package within the R software will be applied to construct the appropriate nomogram. Additionally, the total score for all patients was computed utilizing this nomogram, and two pivotal thresholds for these total scores were determined through X-tile software. Subsequently, non-surgical EGC patients who have undergone chemotherapy were stratified into high-risk, medium-risk, and low-risk subgroups based on these identified critical values.

### Ethical approval

The waiver of ethical permission was justified because the SEER database contains de-identified patient information.

## Results

### Characterization of included cases

In our study, a total of 1879 EGC patients were included from the SEER database. Table 1 provides an overview of their demographic and clinicopathological characteristics, encompassing 1395 (74.24%) male and 484 (25.76%) female cases. Concerning ethnicity, the majority identified as white (N = 1530, 81.43%). Using defined age cutoff values (65–69, 70–76, > 76 years), 748 patients, constituting 39.81% of the population, were aged > 76 years. Simultaneously, employing the optimal cutoff value for tumor size (< 35, 35–71, > 71 m), a predominant proportion of individuals (N = 977, 52.00%) exhibited tumors with diameters of 35–71 mm. Adenocarcinoma (N = 1531, 81.48%) and N1 stage (N = 937, 49.87%) were frequently observed in our study population.

**Table 1:** Demographic and clinicopathological characteristics of non-operated elderly gastric cancer patients who have undergone chemotherapy.

Variables	SEER cohort (n, %)			*p*
	Total N = 1879	Training cohort N = 1316	Validation cohort N = 563	
Marital status				
Married	1276 (67.91)	899 (68.31)	377 (66.96)	0.6027
Unmarried^a^	603 (32.09)	417 (31.69)	186 (33.04)	
Gender				
Female	484 (25.76)	329 (25.00)	155 (27.53)	0.2749
Male	1395 (74.24)	987 (75.00)	408 (72.47)	
Race				
Black	164 (8.73)	116 (8.81)	48 (8.53)	0.3406
Other^b^	185 (9.85)	138 (10.49)	47 (8.35)	
White	1530 (81.43)	1062(80.70)	468 (83.13)	
Age				
65–69	486 (25.86)	354 (26.90)	132 (23.45)	0.2803
70–76	645 (34.33)	448 (34.04)	197 (34.99)	
≥ 77	748 (39.81)	514 (39.06)	234 (41.56)	
Primary site				
Cardia/fundus	1247 (66.37)	850 (64.59)	397 (70.52)	0.0753
Body	287 (15.27)	214 (16.26)	73 (12.97)	
Antrum/pylorus	182 (9.69)	136 (10.33)	46 (8.17)	
Other	163 (8.67)	116 (8.81)	47 (8.35)	
Grade				
I–II	663 (35.28)	458 (34.80)	205 (36.41)	0.5378
III–IV	1216 (64.72)	858 (65.20)	358 (63.59)	
Histology				
Adenocarcinoma	1531 (81.48)	1060 (80.55)	471 (83.66)	0.2558
Other	148 (7.88)	107 (8.13)	41 (7.28)	
Signet ring cell carcinoma	200 (10.64)	149 (11.32)	51 (9.06)	
T stage				
T1–2	1345 (71.58)	946 (71.88)	399 (70.87)	0.696
T3–4	534 (28.42)	370 (28.12)	164 (29.13)	
N stage				
N0	780 (41.51)	538 (40.88)	242 (42.98)	0.143
N1	937 (49.87)	675 (51.29)	262 (46.54)	
N2	121 (6.44)	77 (5.85)	44 (7.82)	
N3	41 (2.18)	26 (1.98)	15 (2.66)	
M stage				
M0	1087 (57.85)	782 (59.42)	305 (54.17)	0.0394
M1	792 (42.15)	534 (40.58)	258 (45.83)	
Chemotherapy				
No/Unknown	22,703 (80.76)	15,912 (80.86)	6791 (80.54)	0.821
Yes	5408 (19.24)	3767 (19.14)	1641 (19.46)	
Radiotherapy				
No	833 (44.33)	592 (44.98)	241 (42.81)	0.4122
Yes	1046 (55.67)	724 (55.02)	322 (57.19)	
Tumor size				
< 35	632 (33.63)	441 (33.51)	191 (33.93)	0.2815
35–71	977 (52.00)	675 (51.29)	302 (53.64)	
≥ 72	270 (14.37)	200 (15.20)	70 (12.43)	

^a^Includes single, separated, widowed, and divorced.

^b^Includes American Indian/Alaska Native and Asian or Pacific Islander.

### Prognostic factors and nomogram in non-operated EGC patients who have undergone chemotherapy

**Figure 2:**
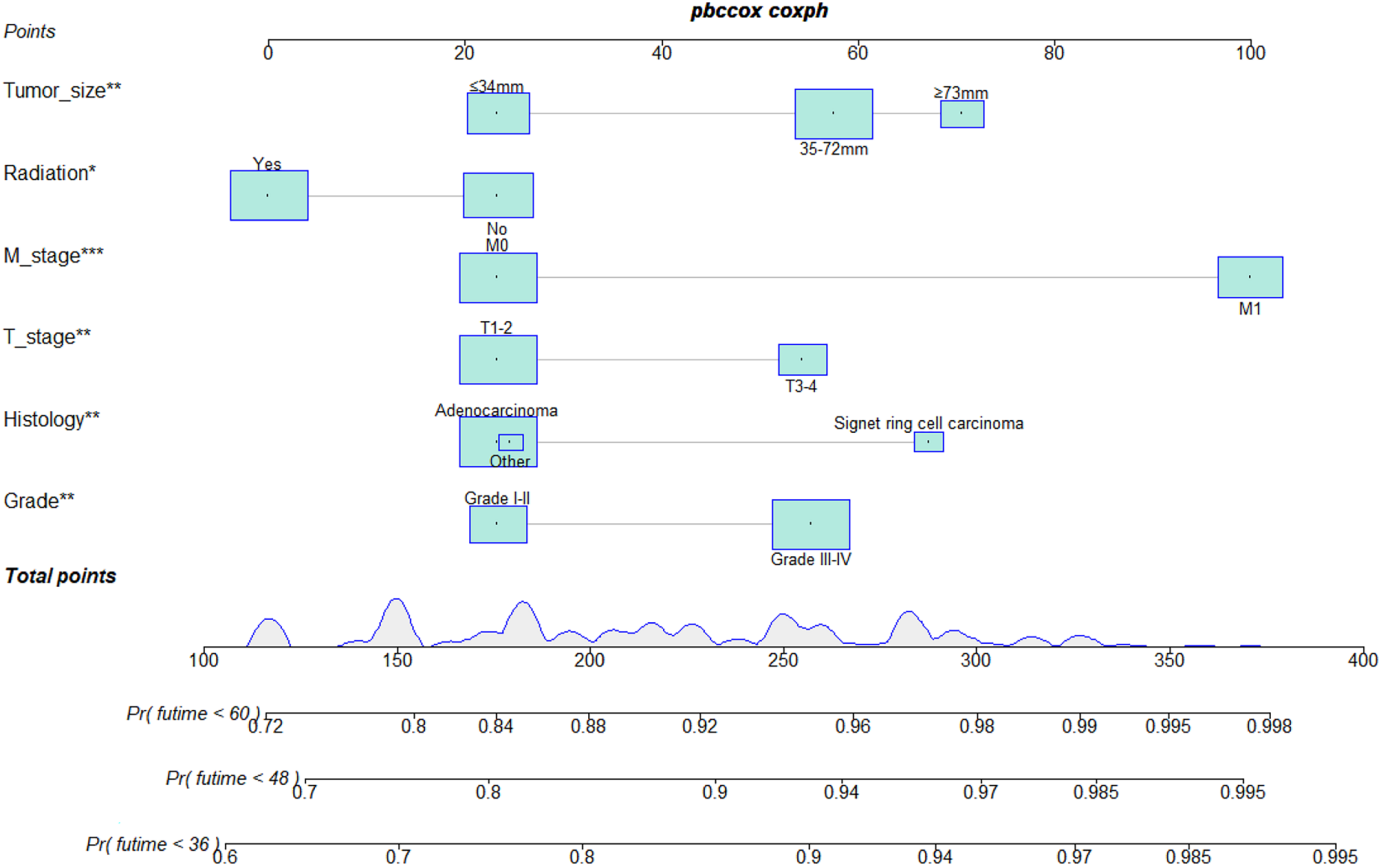
Prognostic nomogram for predicting 36-, 48- and 60 months CS in non-operated EGC patients who have undergone chemotherapy.

The independent prognostic factors for CSS among patients with non-operated EGC who have undergone chemotherapy were identified using univariate and multivariate Cox analyses, including grade, histology, M stage, radiotherapy, tumor size, and T stage (Table 2). A prediction model was developed using these independent prognostic variables to figure out the CSS at 3, 4, and 5 years (Fig. 2).

**Table 2:** Univariate and multivariate COX analysis to determine the independent prognostic factors of non-operated elderly gastric cancer patients who have undergone chemotherapy.

Characteristics	Univariate analysis			Multivariate analysis		
	HR	CI	P	HR2	CI2	P2
Age						
65–69	Reference			Reference		
70–76	0.91	0.78–1.06	0.218	NA	NA	NA
≥ 77	0.94	0.81–1.09	0.389	NA	NA	NA
Grade						
I–II	Reference			Reference		
III–IV	1.39	1.22–1.58	0	1.21	1.06–1.38	0.0058
Histology						
Adenocarcinoma	Reference			Reference		
Other	1.16	0.93–1.45	0.187	1.03	0.82–1.3	0.7715
Signet ring cell carcinoma	1.44	1.19–1.72	0	1.33	1.1–1.61	0.0035
Marital status						
Married	Reference			Reference		
Unmarried^a^	0.97	0.85–1.1	0.604	NA	NA	NA
M						
M0	Reference			Reference		
M1	1.78	1.58–2.01	0	1.59	1.39–1.82	0
N stage						
N0	Reference			Reference		
N1	1.12	0.99–1.27	0.074	1.06	0.93–1.21	0.3685
N2	1.28	0.99–1.66	0.056	1.24	0.96–1.61	0.1065
N3	1.52	1.02–2.28	0.042	1.32	0.87–1.98	0.1898
Primary site						
Cardia/fundus	Reference			Reference		
Body	1.12	0.95–1.32	0.182	0.94	0.79–1.11	0.4518
Antrum/pylorus	1.2	0.99–1.47	0.068	0.97	0.78–1.19	0.76
Other	1.42	1.15–1.75	0.001	1.08	0.87–1.35	0.4838
Race						
Black	Reference			Reference		
Other^b^	1.25	0.95–1.65	0.105	NA	NA	NA
White	1	0.81–1.24	0.997	NA	NA	NA
Radiotherapy						
No	Reference			Reference		
Yes	0.69	0.61–0.77	0	0.85	0.74—0.98	0.0256
Sex						
Female	Reference			Reference		
Male	0.97 0.85–1.11	0.85–1.11	0.688	NA	NA	NA
T stage						
T1–2	Reference			Reference		
T3–4	1.32	1.16–1.51	0	1.18	1.03—1.36	0.0161
Tumor size						
≤ 35 mm	Reference			Reference		
36–71 mm	1.34	1.17–1.53	0	1.22	1.06—1.4	0.0046
≥ 72 mm	1.56	1.29–1.88	0	1.3	1.07—1.57	0.0086

^a^Includes single, separated, widowed, and divorced.

^b^Includes American Indian/Alaska Native and Asian or Pacific Islander.

The nomogram illustrates that each variable corresponds to a specific score on the top horizontal axis, effectively converting the risk associated with each factor into a numerical value. The patient's total score is computed by summing the scores corresponding to each factor. Subsequently, locate this total score on the upper horizontal axis marked with “points,” draw a vertical line downward, and intersect it with the three lower horizontal axes marked with “Pr (futime < 36, 48, 60)” to ascertain the corresponding survival time (CSS).

For example, a 76-year-old married white female patient diagnosed with GC, and she refused surgery, opting for chemotherapy and radiotherapy. The cancer was classified as Grade II, adenocarcinoma, with a TNM stage of T3N1M1 and a tumor size of 3.4 cm. Through the nomogram, we quantify each prognostic factors, and then we get the total score of this patient. According to the total score, we respectively get the probability of death due to GC in three years is 83%, the probability of death due to GC in four years is 86%, and the probability of death due to GC in 5 years is 91%. In the above example, we can use a nomogram to estimate the 3-, 4-, and 5-years survival rates and to further develop a personalized treatment plan. In addition, because of the nomograms' simple and intuitive nature, doctor-patient conflicts caused by forecasting the survival times can be efficiently reduced. Interestingly, this nomogram can also be used as a follow-up guide, thereby making long-term care for patients with EGC relatively more manageable.

**Figure 3:**
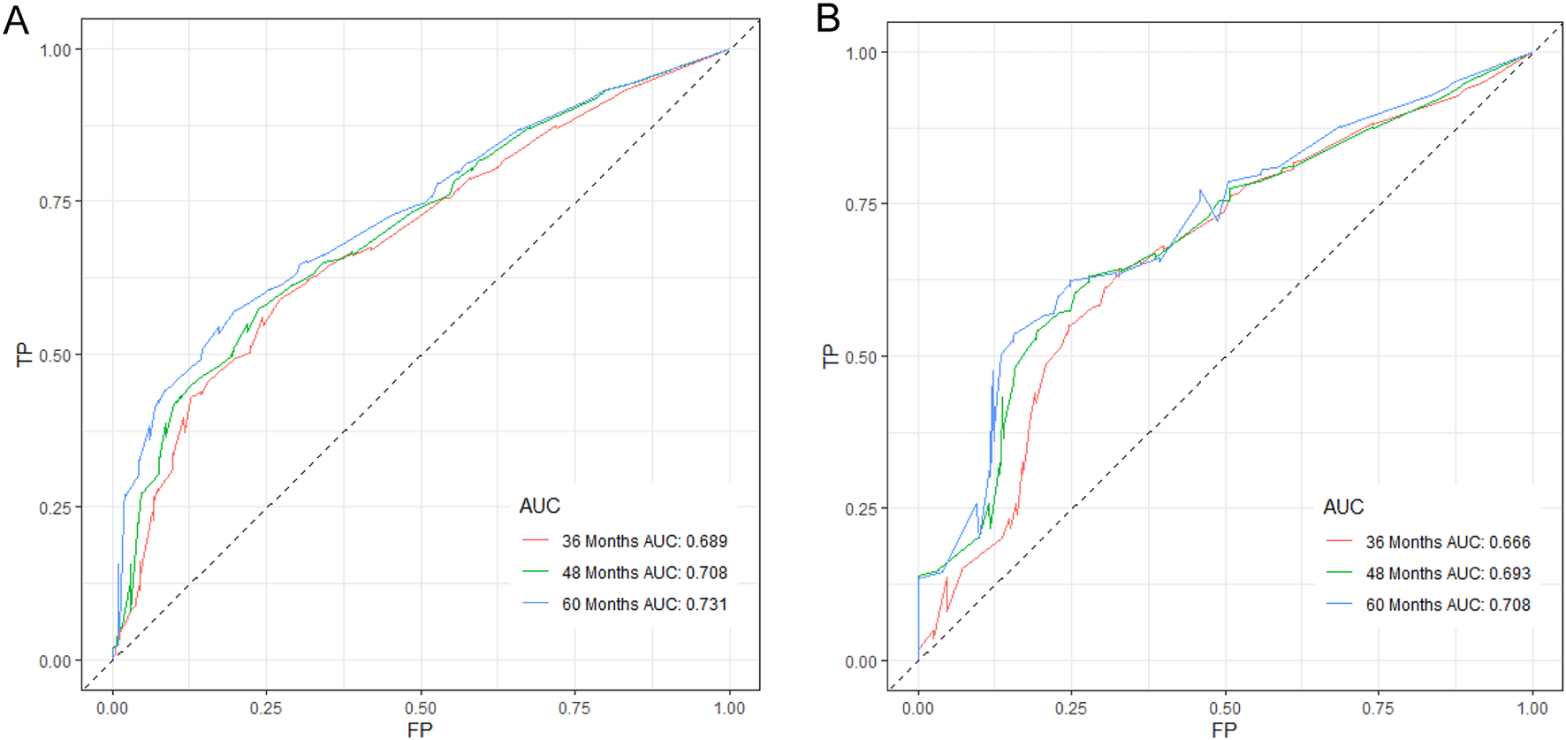
ROC curve of the prognostic nomogram for 36, 48, and 60 months in the training cohort (**A**) and the validation cohort (**B**).

**Figure 4:**
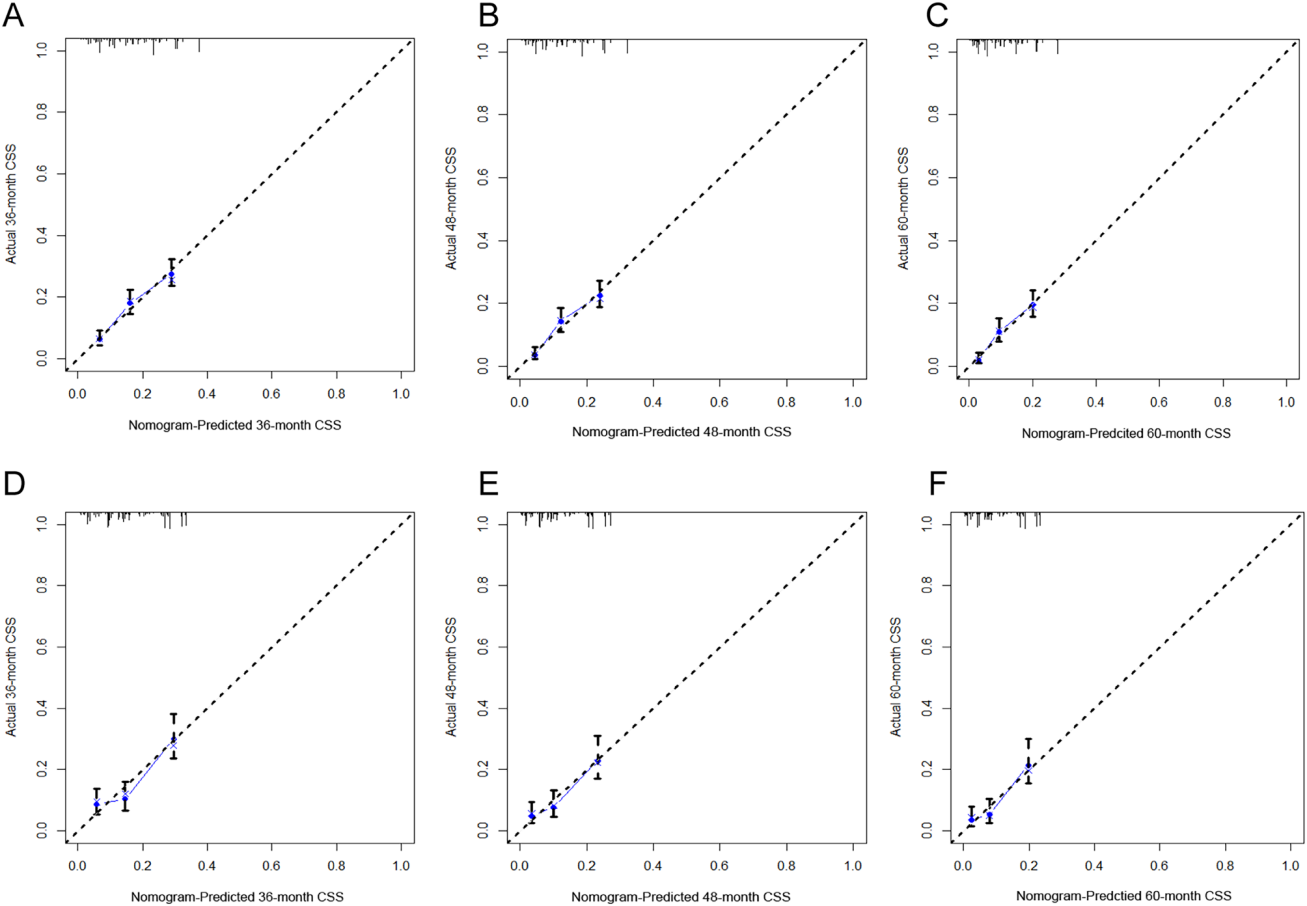
The calibration curves of the prognostic nomogram for the 3 years, 4 years, and 5 years in the training cohort (**A**–**C**) and in the validation cohort (**D**–**F**).

Upon internal validation, we found that the AUC of the prognostic nomogram for the 3-, 4-, and 5-years CSS were 0.689, 0.708, and 0.731 in the training cohort, and 0.666, 0.693, and 0.708 in the validation cohort, respectively, according to the ROC curves (Fig. 3A,B). The horizontal axis of the calibration curve reflected the estimated CSS, whereas the ordinate represented the true CSS. The anticipated and actual curves fit together, thereby indicating that when predicting the CSS of the target population at 3, 4, and 5 years, the nomogram showed a high degree of concordance between the forecast and the real survival in both the training (Fig. 4A–C) and validation cohorts (Fig. 4D–F).

**Figure 5:**
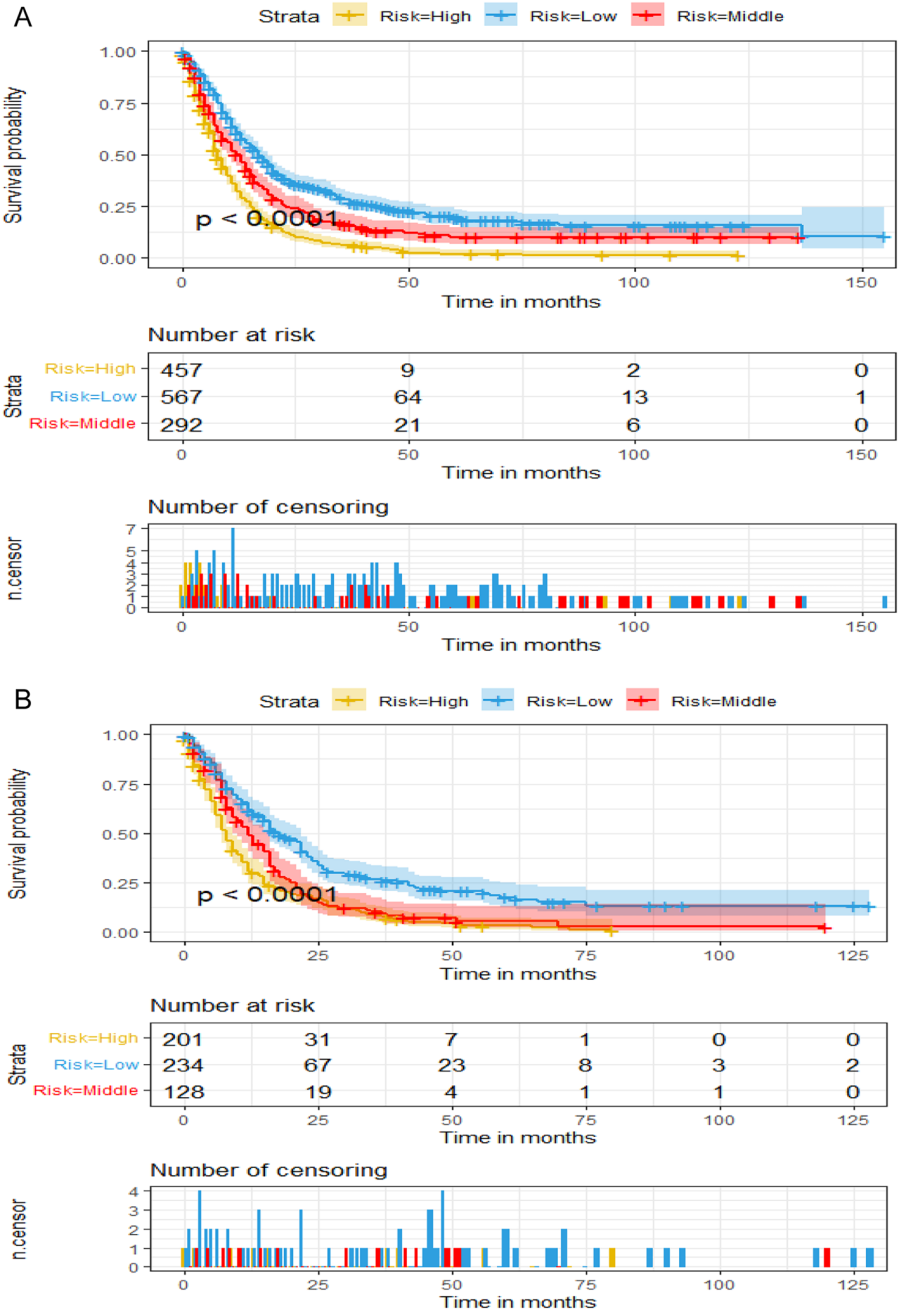
Kaplan–Meier survival curves of three subgroups in the training cohort (**A**) and validation cohort (**B**).

Furthermore, computations were conducted to ascertain the total score for all patients using a prognostic nomogram. Subsequently, we used X-tile software to determine two threshold points for all patient scores, which were used to separate the recipients into three distinct groups for Kaplan-Meier survival analysis (Fig. 5A,B). As illustrated in Fig. 5, patients in the high-risk group (score > 247) experienced considerably inferior survival outcomes as compared to those in the median-risk group (201–247) and low-risk group (score < 201).

## Discussion

The incidence and mortality rates of GC escalate with advancing age^14^. However, clinical investigations and therapeutic interventions for GC have not comprehensively addressed the distinctive needs of elderly individuals, who represent the predominant demographic affected by this disease^15^. Moreover, the clinical characteristics of this demographic are highly intricate^16^. Firstly, a considerable number of elderly patients are unable to withstand invasive screening methods due to weakened physical conditions, including heart disease, respiratory diseases, cerebrovascular diseases, and so forth^17^. Furthermore, owing to the absence of evident symptoms in the early stages of GC, the disease commonly advances to the late stage by the time of initial diagnosis, consequently leading to missed opportunities for optimal surgical intervention. Secondly, some factors have hindered the surgical treatment of EGC, including various potential comorbidities, organ dysfunction, decreased immune function, and poor treatment willingness^18^. Finally, considering that elderly patients are more prone to serious post-operative complications, surgeons are less inclined to pursue surgical treatments^4,19^. From the perspective of this special population, non-surgical treatments, such as chemotherapy, are the most appropriate options.

Currently, there is a consensus that the most effective methods for improving the CSS of patients with GC involve the implementation of preventive measures and the provision of personalized treatment strategies^20^. Developing individualized treatment strategies for non-operated EGC patients who have undergone chemotherapy requires clinicians to accurately predict the CSS by integrating tumor and patient clinical characteristics to assess whether the treatment is excessive or insufficient. However, few studies have identified this important subset of patients nor have explored their prognoses. Instead, the vast majority of studies have concentrated on young individuals or those with GC who have undergone surgery and their prognoses^21–24^. Patients who did not undergo surgery but had been administered chemotherapy were the main subjects of this study, a major breakthrough in the field of GC research. we identified independent prognostic factors and developed a nomogram for predicting the CSS in non-operated patients with EGC who have undergone chemotherapy.

The present study identified six independent prognostic factors for CSS in patients with non-operated EGC who have undergone chemotherapy. These variables included tumor grade, histology, M stage, radiotherapy, tumor size, and T stage.

In general, the development pattern of primary GC encompasses perpendicular growth concerning the stomach wall as well as horizontal growth along the gastric wall; the former refers to the depth of tumor infiltration (T stage) in GC, while the latter refers to the size of the tumor^25^. T stage consistently emerges as an independent risk factor influencing the prognosis of cancer patients. Undoubtedly, the outcomes of this study reaffirm the pivotal role of T stage as a significant prognostic factor for individuals with EGC who have undergone chemotherapy without received surgical intervention. Regarding solid organs, such as lung and breast cancers, the concept of T staging has been described in the assessment of tumor size^26,27^. However, the prognostic implications of tumor size in GC have not been established. Previous studies have demonstrated a correlation between tumor size and GC prognosis^28,29^. In the present study, tumor size was identified as an independent prognostic factor in this cohort of elderly patients. There are some reasonable explanations for this phenomenon: larger tumors have a deeper invasion and a heightened possibility of lymph node metastasis. Meanwhile, the existence of larger tumors may indicate a worsened tumor burden, thus increasing the difficulty of administering chemotherapy to older patients who are frail and not candidates for surgery.

Gastric signet ring cell carcinoma is a distinct GC subtype characterized by limited cellular differentiation, heightened aggressiveness, and expedited disease progression^30^. The findings of our study indicate that the presence of signet ring cell carcinoma serves as an independent prognostic factor in patients with non-operated EGC who have undergone chemotherapy. This can be attributed to two factors. Firstly, numerous studies have provided empirical evidence supporting the notion that gastric signet ring cell carcinoma exhibits a significantly elevated rate of peritoneal metastasis as compared with other tumor forms, which can increase the risk of mortality in EGC patients^31,32^. Secondly, as reported by Lemoine et al.^33^, the effectiveness of chemotherapy in addressing gastric signet ring cell carcinoma was regarded as less than ideal, as reflected in a median survival time of 5.6 months. On the other hand, non-signet ring cell carcinoma exhibited a comparatively more favorable response to chemotherapy, with a median survival period of 9.4 months^33^. In the context of older patients diagnosed with gastric signet ring cell carcinoma, there is a pressing need for further investigation to establish tailored and specialized therapeutic strategies with the goal of extending survival and improving the quality of life for these patients.

The TNM staging system, which serves to assess patient prognosis and to devise appropriate treatment strategies, is the predominant method employed for the clinical staging of GC^34^. When classified as M1, this indicated that distant metastasis has occurred. GC has the ability to undergo metastasis by various mechanisms, including lymph node, hematogenous, direct, and implantation metastases^35^. Lymphatic metastasis is the dominant route of spread, whereas hematogenous metastasis is more likely to result in patient mortality^35^. Previous studies have demonstrated that the presence of distant-site metastases resulted in a significant survival time reduction^36,37^. Unfortunately, inconspicuous symptoms of early GC contribute to a higher incidence of diagnoses at the M1 stage, with approximately 40% displaying distant metastasis, leading to an unfavorable prognosis characterized by a median CSS period of 10 months and a 5 years survival rate ranging only from 3 to 6%^38,39^. Our findings indicated that M1 is an independent prognostic factor in EGC patients who undergo non-surgical management with chemotherapy.

Previous studies on EGC have shown that age, N stage, and primary site are significant prognostic variables for patients with EGC^40^. After imposing restrictions on the selection of treatment modalities for the elderly population and focusing solely on the prognostic outcomes of those who received chemotherapy without surgical intervention, the multivariate COX regression analysis revealed that the aforementioned components did not possess significant independent prognostic value within our study population. This occurrence not only implied considerable disparities in their prognosis but also stressed the significance of individual evaluation.

Nonetheless, our predictive model is not without its shortcomings. Firstly, given the retrospective nature of our investigation, additional prospective multicenter studies are required to validate our findings. Secondly, the SEER database contained incomplete and ambiguous information. For instance, several pivotal variables, including details on regimens and cycles of chemotherapy, targeted therapy, and immunological therapy, as well as complications such as perforation, bleeding, and obstruction, and post-chemotherapy complications, were notably absent. Finally, due to the scarcity of patients in our medical center meeting the inclusion criteria for this study, an external validation of the predictive model was not undertaken. In subsequent research efforts, it will be imperative to gather cases from various medical centers worldwide, thereby enhancing the comprehensiveness and generalizability of our study.

## Conclusion

The current study successfully identified independent predictive factors associated with CSS in elderly patients with unresected GC who have received chemotherapy, including grade, histology, M stage, radiotherapy, tumor size, and T stage. A nomogram was developed using these independent prognostic variables to figure out the CSS at 3, 4, and 5 years. The implementation of this nomogram may harbor the potential to aid surgeons in developing personalized treatment strategies targeting this specific patient population.

## Data Availability

Publicly available datasets were analyzed in this study. These data can be found in the SEER database (Incidence-SEER 18 Regs Customs Data).
